# Long-Term Fluorescent Tissue Marking Using Tissue-Adhesive Porphyrin with Polycations Consisting of Quaternary Ammonium Salt Groups

**DOI:** 10.3390/ijms23084218

**Published:** 2022-04-11

**Authors:** Yoshiki Komatsu, Toru Yoshitomi, Kinji Furuya, Takafumi Ikeda, Azusa Terasaki, Aoi Hoshi, Naoki Kawazoe, Guoping Chen, Hirofumi Matsui

**Affiliations:** 1 Graduate School of Comprehensive Human Sciences, University of Tsukuba, 1-1-1 Tennodai, Tsukuba, Ibaraki 305-8577, Japan; s0611608@yahoo.co.jp (Y.K.); tikeda218@gmail.com (T.I.); aotagiri@gmail.com (A.H.); 2 Research Center for Functional Materials, National Institute for Materials Science, 1-1 Namiki, Tsukuba, Ibaraki 305-0044, Japan; kawazoe.naoki@nims.go.jp (N.K.); guoping.chen@nims.go.jp (G.C.); 3 Department of Gastrointestinal and Hepato-Biliary-Pancreatic Surgery, Faculty of Medicine, University of Tsukuba, 1-1-1 Tennodai, Tsukuba, Ibaraki 305-8575, Japan; kfuruya@md.tsukuba.ac.jp; 4 Department of Breast-Thyroid-Endocrine Surgery, University of Tsukuba Hospital, 2-1-1 Amakubo, Tsukuba, Ibaraki 305-8576, Japan; azyu0713@gmail.com; 5 Division of Gastroenterology, Faculty of Medicine, University of Tsukuba, 1-1-1 Tennodai, Ibaraki 305-8575, Japan

**Keywords:** polycation, polyamine, porphyrin, tissue marking, tissue adhesive

## Abstract

Localization of tumors during laparoscopic surgery is generally performed by locally injecting India ink into the submucosal layer of the gastrointestinal tract using endoscopy. However, the location of the tumor is obscured because of the black-stained surgical field and the blurring caused by India ink. To solve this problem, in this study, we developed a tissue-adhesive porphyrin with polycations consisting of quaternary ammonium salt groups. To evaluate the ability of tissue-adhesive porphyrin in vivo, low-molecular-weight hematoporphyrin and tissue-adhesive porphyrin were injected into the anterior wall of the exposed stomach in rats. Local injection of low-molecular-weight hematoporphyrin into the anterior wall of the stomach was not visible even after 1 day because of its rapid diffusion. In contrast, the red fluorescence of the tissue-adhesive porphyrin was visible even after 7 days due to the electrostatic interactions between the positively-charged moieties of the polycation in the tissue-adhesive porphyrin and the negatively-charged molecules in the tissue. In addition, intraperitoneal injection of tissue-adhesive porphyrin in rats did not cause adverse effects such as weight loss, hepatic or renal dysfunction, or organ adhesion in the abdominal cavity. These results indicate that tissue-adhesive porphyrin is a promising fluorescent tissue-marking agent.

## 1. Introduction

Laparoscopic surgery for gastrointestinal cancer has been widely performed since it was first reported in the 1980s [1]. Laparoscopic surgery is less invasive than open surgery, which significantly improves the patient’s quality of life. In addition, most recently, innovative technologies such as robotic-assisted laparoscopic surgery have been developed and used in hospitals. It is not only more precise, but is also a more non-invasive surgery, further improving the patient’s quality of life [2]. Since localization of tumors cannot be identified from the serosal side during laparoscopic surgery, preoperative tissue marking is conducted using endoscopy.

For preoperative tissue marking, India ink, first reported in 1975 by Ponsky and King [3], has been conventionally used in hospitals. After India ink is injected into the submucosal layer around the tumor using the endoscope, the tumor tissue is removed by laparoscopic surgery according to the position of the marking with India ink. However, the black ink diffuses in the tissue to expand the tissue marking area owing to its non-adhesive property, leading to difficulties in recognizing the cancer site and subsequent inappropriate resection of the normal tissue [4,5,6,7]. In addition, various complications associated with India ink have been reported, including focal peritonitis, inflammatory pseudotumor, abscess, and postoperative adhesion ileus [8,9,10,11,12,13,14]. The reason for this is that India ink leaked into the abdominal cavity remains permanently, which causes inflammation. India ink cannot be eliminated if it spills out of the serosa, which is a disadvantage when performing laparoscopic surgery [7].

To solve the problems of India ink, we focused on porphyrin derivatives as fluorescent marking agents. Porphyrins are heterocyclic macrocycle organic compounds with a large Stokes shift between absorption (~400 nm) and red emission [15], which are visible in vivo without a specific fluorescent detector. Unlike the black color of India ink, the fluorescence signal of porphyrin enables us to visualize the exact localization of the tumor during surgery; the fluorescence of porphyrin has been used for the detection of tumors and image-guided surgery [16,17]. However, low-molecular-weight porphyrin derivatives rapidly diffuse after local injection into tissues. Such a short marking ability of low-molecular-weight porphyrin limits the surgical schedule, and surgery must be conducted immediately. Thus, development of long-term marking agents with porphyrins would make it easier to schedule surgery. To accomplish the long-term fluorescent tissue marking, in this study, a copolymer of poly[2-(methacryloyloxy)ethyl]trimethylammonium chloride and poly[*N*-(3-aminopropyl)methacrylamide hydrochloride] conjugated with hematoporphyrin (HpD), PMETAC-*co*-PAPMAA(HpD), was developed as a tissue-adhesive marking agent. Glycosaminoglycans (GAGs), such as chondroitin sulfate, heparan sulfate, and hyaluronan, are large linear polysaccharides composed of repeating disaccharide units with highly negatively-charged carbohydrate side chains and are abundantly present in the submucosa layer [18]. Owing to the multi-valent electrostatic interactions with negatively-charged macromolecules, the polycation moieties, PMETAC, may be retained for a longer period of time after local injection into the submucosal layer. However, there have been no reports on tissue-marking agents using polycation-based materials. In this study, we evaluated the usefulness and safety of PMETAC-*co*-PAPMAA(HpD) in rats.

## 2. Results and Discussion

### 2.1. Synthesis of Tissue-Adhesive Porphyrin

**Figure 1:**
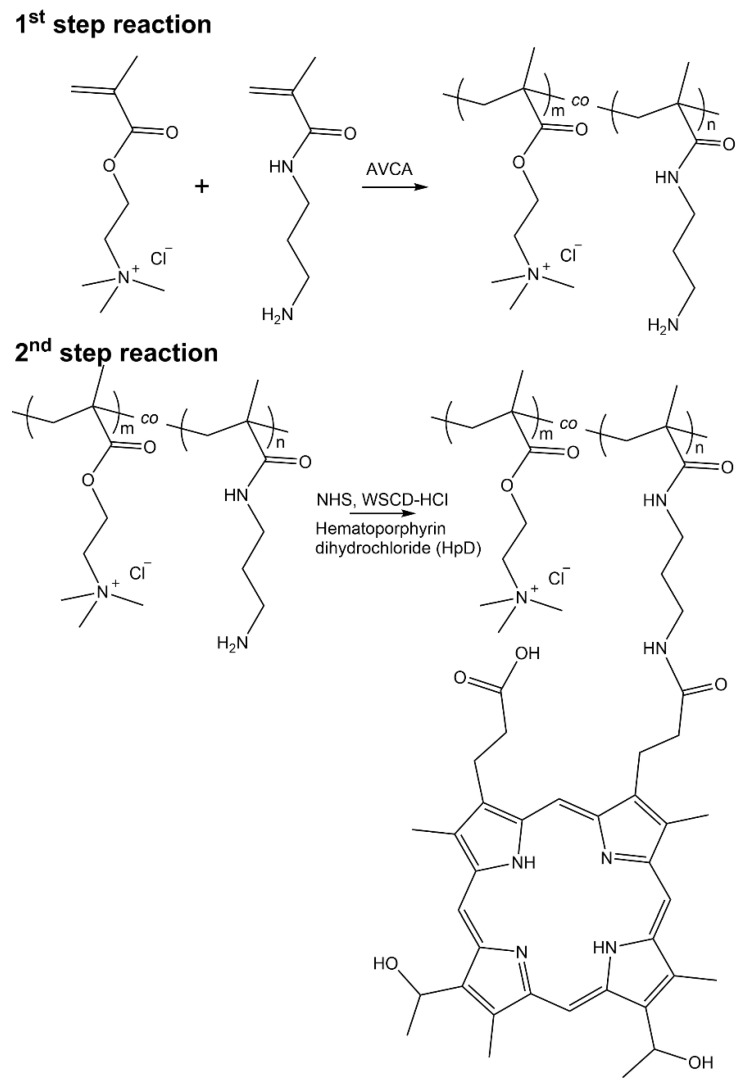
Synthesis scheme of PMETAC-*co*-PAMPAA(HpD).

First, [2-(methacryloyloxy)ethyl]trimethylammonium chloride (METAC) was copolymerized with *N*-(3-aminopropyl)methacrylamide hydrochloride (APMAA) via free-radical polymerization (see Figure 1, 1st step reaction).

**Figure 2:**
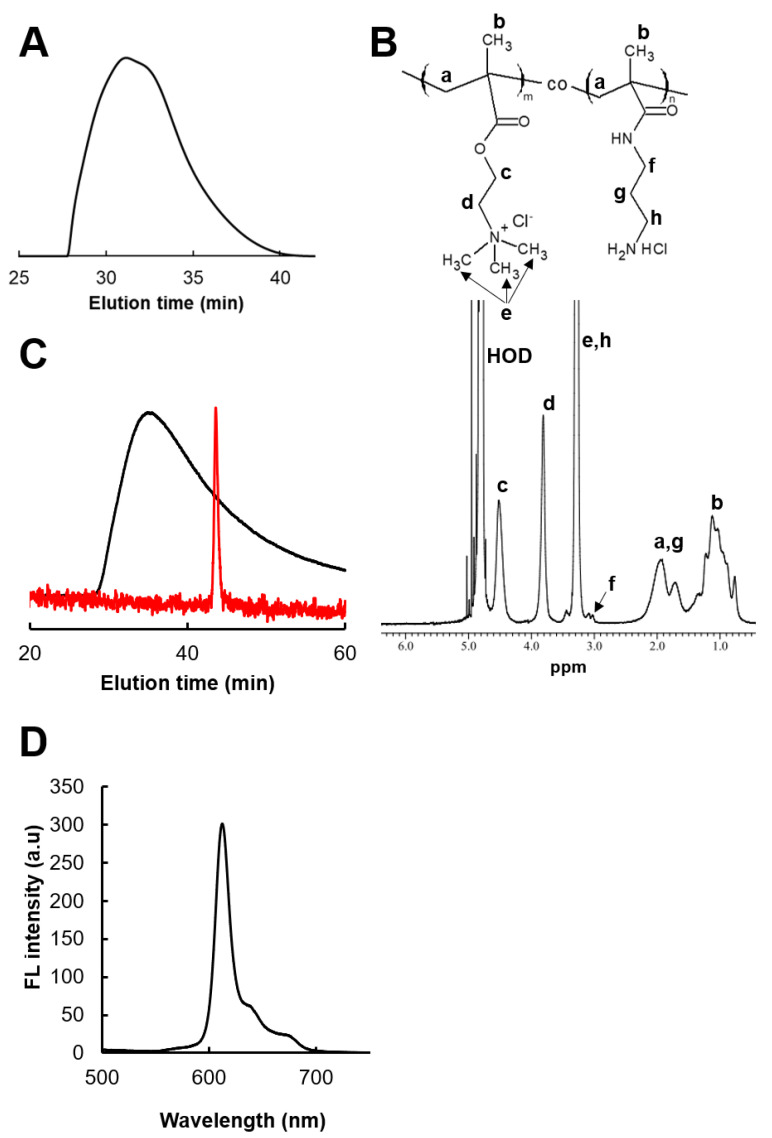
**Characterization of PMETAC-*co*-PAMPAA and PMETAC-*co*-PAMPAA(HpD).** (**A**) SEC chromatogram of PMETAC-*co*-PAMPAA. (**B**) Chemical structure and ^1^H NMR spectrum of PMETAC-*co*-PAMPAA. The lowercase letters in the chemical structure, which show the proton, correspond to the peak in the ^1^H NMR spectrum. (**C**) SEC chromatogram of PMETAC-*co*-PAMPAA(HpD) (black) and HpD (red), recorded by RF-10A_XL_ fluorescence detector at an excitation/emission of 400/635 nm. (**D**) Fluorescent spectrum of PMETAC-*co*-PAMPAA(HpD) by the excitation at 375 nm.

From the size exclusion chromatography (SEC) profile, the number- and weight-average molecular weights of the obtained polymer, PMETAC-*co*-PAPMAA, were 23,500 and 54,500, respectively, and its polydispersity index (PDI) was 2.32 (Figure 2A).

The average unit numbers of PMETAC and PAPMAA in PMETAC-*co*-PAPMAA were 200 and 2, respectively, as determined by ^1^H NMR spectroscopy (Figure 2B). HpD was conjugated with the primary amines in the PMETAC-*co*-PAPMAA copolymer to obtain PMETAC-*co*-PAPMAA(HpD) (see Figure 1, 2nd step). To check the covalent conjugation of hematoporphyrin to the polymer and its purification, SEC was carried out with the fluorescent detector by excitation at 400 nm and emission at 635 nm (Figure 2C). The PMETAC-*co*-PAPMAA(HpD) copolymers were eluted with peak tailing after 30 min (Figure 2C). On the contrary, low-molecular-weight HpD eluted with a sharp peak at 43 min (Figure 2C). The SEC chromatogram of PMETAC-*co*-PAPMAA(HpD) showed no peaks at 43 min. These results indicate the covalent conjugation of hematoporphyrin to the PMETAC-*co*-PAPMAA copolymer and the removal of unreacted HpD by purification with dialysis. To determine the amount of HpD in PMETAC-*co*-PAPMAA(HpD), the absorption spectrum of the PMETAC-*co*-PAPMAA copolymer was measured. From the standard curve of HpD absorbance at 400 nm, it was confirmed that approximately one HpD was introduced into approximately one molecule of PMETAC-*co*-PAPMAA. The introduction of a small amount of HpD is important because introducing a large amount of porphyrin into a polymer chain causes intra- and inter-molecular interactions by π-π stacking of porphyrin, which leads to fluorescence quenching and polymer aggregation [15]. The fluorescent spectrum of PMETAC-*co*-PAPMAA(HpD) showed red porphyrin fluorescence under UV irradiation at 375 nm (Figure 2D).

### 2.2. Cytotoxicity of Tissue-Adhesive Porphyrin

**Figure 3:**
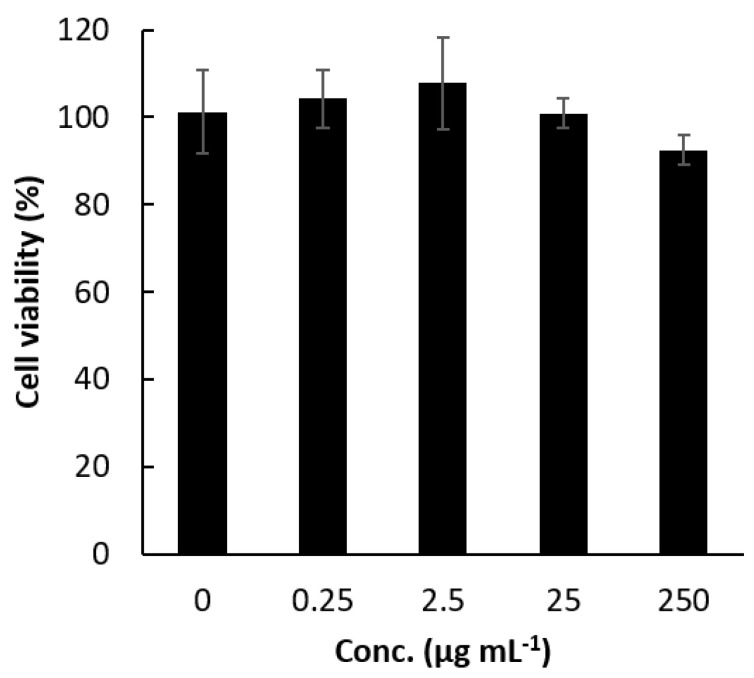
**Cytotoxicity of PMETAC-*co*-PAMPAA(HpD) against RGM-1 cells, which is the gastric epithelial cell line**. Data are shown as mean ± standard deviation (*n* = 4).

Polycations such as polyethyleneimine are known to exhibit high cytotoxicity. Here, the cytotoxicity of PMETAC-*co*-PAPMAA(HpD) against RGM-1 cells, which is a gastric epithelial cell line [19], was evaluated using the WST-8 assay. Even at a concentration of 10 µM (250 µg mL^−1^) of PMETAC-*co*-PAPMAA(HpD), cell viability did not decrease (Figure 3). Bertrand et al. reported that the 50% inhibitory concentration (IC_50_) of linear polyethyleneimine with a molecular weight of 50,000 ranged from 6.5 to 30 μg mL^−1^ [20]. In addition, it has been reported that the IC_50_ of branched polyethyleneimine with a molecular weight of 25,000 was 3.5 μg mL^−1^ [21]. Compared with the reported cytotoxicity of polyethyleneimine, the cytotoxicity of PMETAC-*co*-PAPMAA(HpD) was extremely low.

### 2.3. Tissue Marking Using Tissue-Adhesive Porphyrin

**Figure 4:**
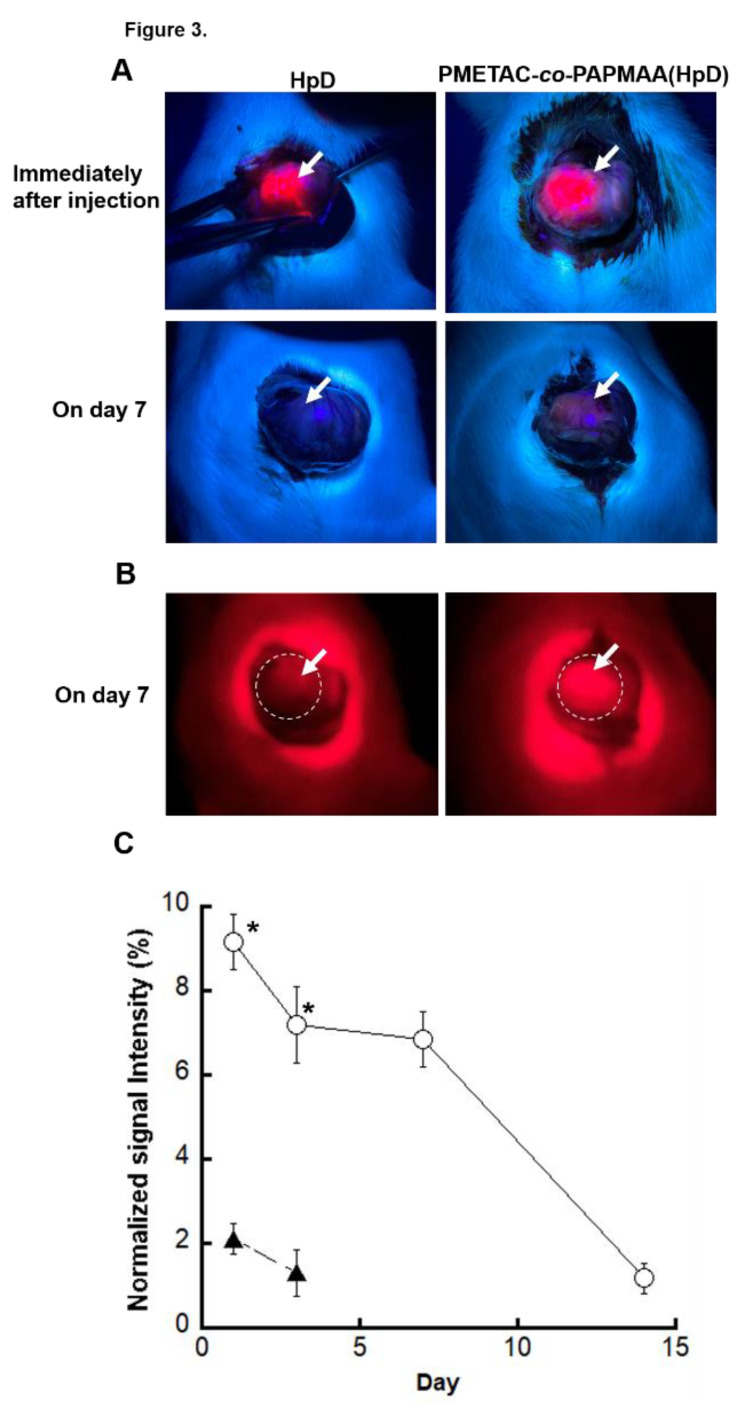
**Fluorescent detection of HpD and PMETAC-*co*-PAMPAA(HpD) on the exposed stomach after their local injection into the anterior wall of the stomach of rats.** (**A**) Photo of the exposed stomach under the irradiation at 375 nm by LED light immediately (upper images) and on day 7 (lower images) after their local injection. White arrows indicate the injection site. (**B**) Photo of the exposed stomach under the irradiation at 375 nm by LED light on day 7 after their local injection, which was captured by digital camera equipped with a red gelatin filter. The fluorescent signals of HpD and aHpD were indicated in the white dotted circle. White arrows indicate the injected site. (**C**) Time course of fluorescent intensity of HpD (closed triangle) and PMETAC-*co*-PAMPAA(HpD) (open circle) on the anterior wall of the extracted stomach. Data are shown as mean ± SEM (*n* = 3). * *p* < 0.05, compared with the HpD treatment.

To investigate the tissue adhesion property of PMETAC-*co*-PAPMAA(HpD) in the living tissue of the gastrointestinal tract, PMETAC-*co*-PAPMAA(HpD) or HpD was locally injected into the anterior wall of the stomach of rats. As shown in Figure 4A (upper images), the red fluorescence of PMETAC-*co*-PAPMAA(HpD) and HpD on the exposed stomach was observed under irradiation at 375 nm by LED light immediately after local injection. However, when low-molecular-weight HpD was administered, red fluorescence was not observed even at 1 day after the local injection (data not shown). In contrast, the visible red fluorescence of PMETAC-*co*-PAPMAA(HpD) was observed even after 7 days of local injection (Figure 4A,B). However, after 14 days, the fluorescence of PMETAC-*co*-PAPMAA (HpD) was not visible (data not shown). Fluorescence intensity in the anterior wall of the stomach was quantified using ImageJ after opening the extracted stomach (Figure S2). One day after local injection, only approximately 10% of the fluorescence intensity remained. Approximately 7% of the injected PMETAC-*co*-PAPMAA(HpD) remained in the stomach tissue even at 7 days after local injection in the anterior wall of the stomach of rats. Thus, the PMETAC-*co*-PAPMAA(HpD) locally injected in the anterior wall of the stomach of rats was able to remain for at least one week due to its tissue adhesion caused by its multi-valent electrostatic interaction. On the other hand, locally injected PMETAC-*co*-PAPMAA(HpD) gradually dissociated from the tissues and eventually diffused throughout the body. This gradual dissociation of PMETAC-*co*-PAPMAA(HpD) from tissues is probably due to the interaction with various negatively-charged substances, such as proteins in the tissue.

### 2.4. Safety of Tissue-Adhesive Porphyrin after Intraperitoneal Injection

**Figure 5:**
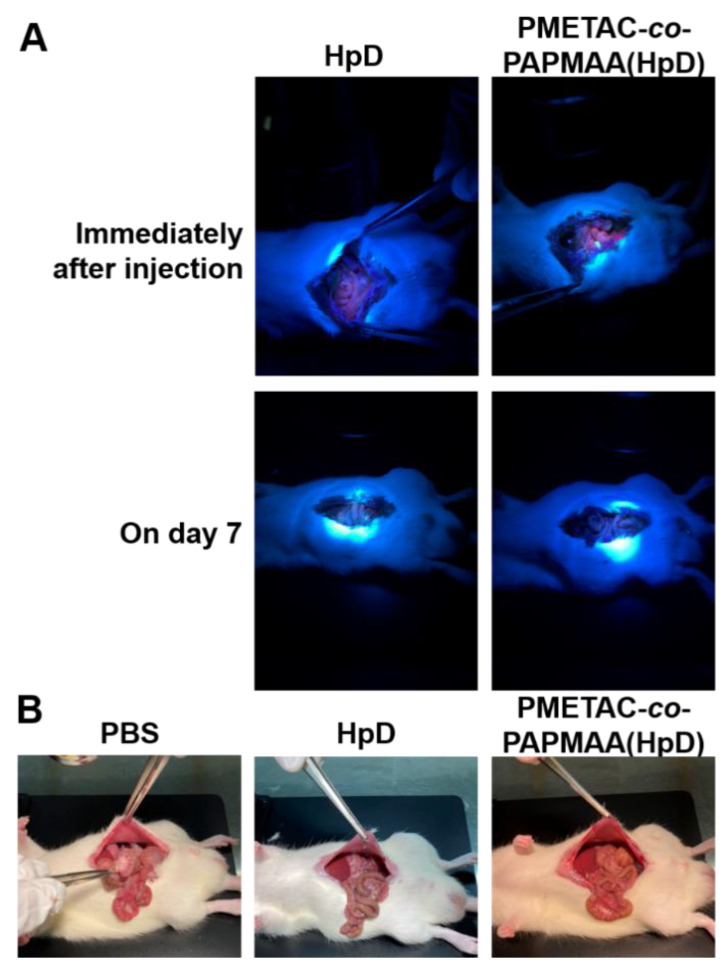
**Fluorescent detection and confirmation of tissue adhesion after intraperitoneal injection of HpD and PMETAC-*co*-PAMPAA(HpD)**. (**A**) Fluorescent detection of HpD and PMETAC-*co*-PAMPAA(HpD) on day 7 after intraperitoneal injection. (**B**) Abdominal tissue adhesion on day 7 after intraperitoneal injection of PBS, HpD, and PMETAC-*co*-PAMPAA(HpD).

Since the locally injected PMETAC-*co*-PAPMAA(HpD) eventually diffused throughout the body, the safety of PMETAC-*co*-PAPMAA(HpD) was investigated. An amount of 100 µL of 5 mg/mL PMETAC-*co*-PAPMAA(HpD) or 200 µM HpD was injected intraperitoneally into rats. After injection, the red fluorescence signal of both HpD and PMETAC-*co*-PAPMAA(HpD) was visible after irradiation at 375 nm (Figure 5A). In contrast, the red fluorescence signals of both HpD and PMETAC-*co*-PAPMAA(HpD) were not visible on day 7 after injection. As PMETAC-*co*-PAPMAA(HpD) has positive charges, it may have the potential to cause tissue adhesion between the intestinal tract and the peritoneum. At 7 days after intraperitoneal injection, however, there was no adhesion between the intestinal tract and peritoneum (Figure 5B). As the metabolic pathway of aHpD has not yet been clarified, we would like to investigate the biodistribution of aHpD in the near future.

**Figure 6:**
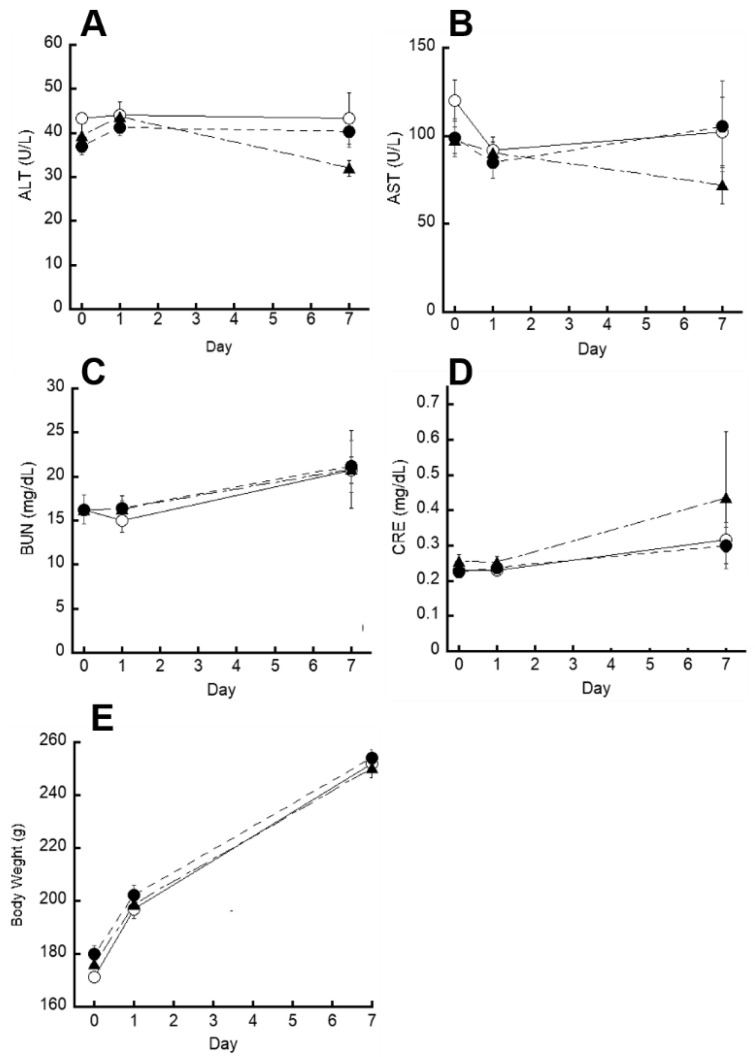
**Acute toxicity of PMETAC-*co*-PAMPAA(HpD).** The comparison of plasma (**A**) ALT, (**B**) AST, (**C**) BUN and (**D**) CRE between test group and control group after intraperitoneal injection of PBS (open circle), HpD (closed triangle), PMETAC-*co*-PAMPAA(HpD) (closed circle). ALT: alanine aminotransferase, AST: aspartate aminotransferase, BUN: blood urea nitrogen, CRE: creatinine. (**E**) Changes in body weight after intraperitoneal injection of PBS (open circle), HpD (closed triangle), PMETAC-*co*-PAMPAA(HpD) (closed circle). Data are shown as mean ± SEM (*n* = 3).

To investigate the side effects of intraperitoneal injection of PMETAC-*co*-PAPMAA(HpD), the plasma levels of alanine aminotransferase (ALT), aspartate aminotransferase (AST), blood urea nitrogen (BUN), and creatinine (CRE) were measured to evaluate liver and kidney toxicity (Figure 6A–D). The results of these damage markers showed no obvious fluctuations compared to PBS, indicating no damage or disruption to liver and renal functions. In addition, the body weight of the rats did not decrease compared to that of the control (Figure 6E). This indicates that the gastrointestinal tract worked well even after local injection of PMETAC-*co*-PAPMAA(HpD) into the rat stomach wall, implying that there was no gastrointestinal damage. These results indicated the high safety of PMETAC-*co*-PAPMAA(HpD). Since the molecular weight of PMETAC-*co*-PAPMAA (HpD) is approximately 20 kDa, even if it leaks excessively into the abdominal cavity, it would be absorbed and renally excreted.

### 2.5. Visuality of PMETAC-co-PAPMAA(HpD) after Local Injection into the Colon of Excised Pigs

**Figure 7:**
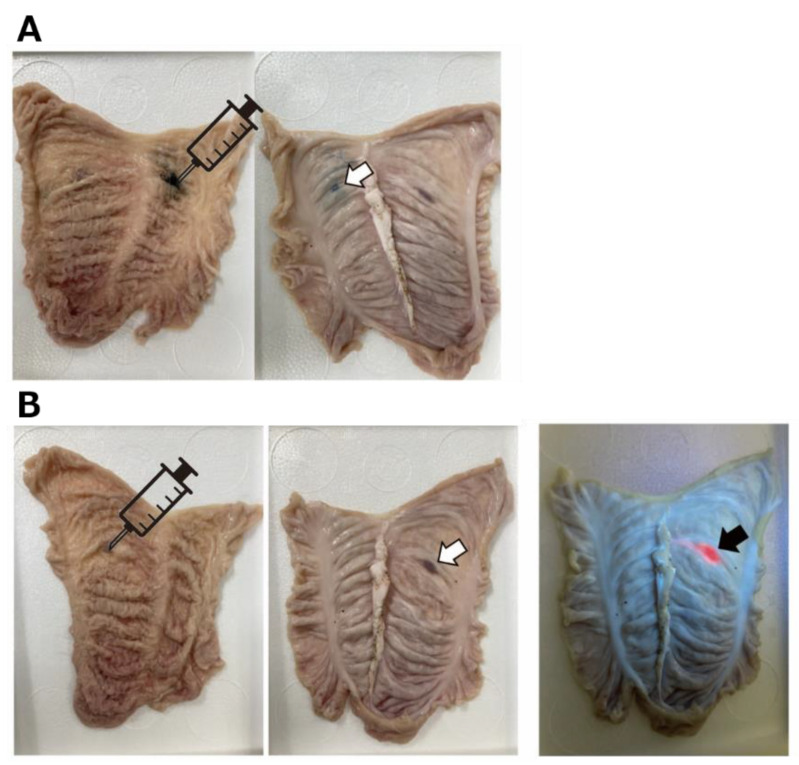
**The photographs showing the marking of (A) India ink and (B) PMETAC-*co*-PAMPAA(HpD) in the porcine colon tissue.** (**A**) (**left**) India ink was injected from mucosal sides. The illustrations of syringes indicate the location of injection site. (**right**) The photographs from the serosal side (outside of the digestive tract) after local injection. White arrows indicate the location of injection site. (**B**) (**left**) PMETAC-*co*-PAMPAA(HpD) was injected from mucosal sides. The illustrations of syringes indicate the location of injection site. (**middle**) The photographs from the serosal side (outside of the digestive tract) after local injection. White arrows indicate the location of injection site. (**right**) The photographs from the serosal side under the irradiation at 375 nm. Black arrow indicates the strong fluorescent signal of injection site.

To evaluate visibility, India ink or PMETAC-*co*-PAPMAA(HpD) was injected into the submucosal layer of the excised porcine colon (Figure 7). When observed from the serosal side using India ink, it was difficult to discern where the border of the marking agent was located. In the absence of UV irradiation, tissue marking of PMETAC-*co*-PAPMAA(HpD) was similar to that of India ink, whereas the red fluorescence of PMETAC-*co*-PAPMAA(HpD) was clearly observed under UV irradiation at 375 nm. Thus, the visible red fluorescence of porphyrin could be used for tissue marking in the gastrointestinal tract.

Recently, near-infrared (NIR) fluorescent marking methods have been studied and developed using indocyanine green (ICG) [7]. However, low-molecular-weight ICG has rapid diffusion and short longevity after local injection into tissues [7]. Therefore, surgery should be performed as soon as possible after marking to avoid diffusion. In addition, the detection of NIR fluorescence not only requires expensive detectors, but also reduces the resolution owing to the long wavelength. On the other hand, since red fluorescence of porphyrin can be detected by the naked eye, it does not require a specific detector. Therefore, porphyrin-based marking agents can be used in hospitals without advanced medical equipment. To the best of our knowledge, this is the first study to use tissue-adhesive fluorescent polymer materials with porphyrin for long-term tissue marking. Further improvement of the long-term marking ability can be expected by optimizing the molecular weight of tissue-adhesive porphyrin.

## 3. Materials and Methods

### 3.1. Materials

4,4′-Azobis(4-cyanovaleric acid) (ACVA), METAC, and APMAA were purchased from Sigma-Aldrich (St Louis, MO, USA) and were used without purification. Acetic acid, tetramethylammonium hydroxide pentahydrate, and *N*-hydroxysuccinimide were purchased from FUJIFILM Wako Pure Chemical (Chuo-Ku, Osaka, Japan), and 1-ethyl-3-(3-dimethylaminopropyl)-carbodiimide hydrochloride (WSCD-HCl) was purchased from Peptide Institute, Inc. (Ibaraki, Osaka, Japan). HpD was purchased from MedChem Express (San Diego, CA, USA).

### 3.2. Preparation of PMETAC-co-PAPMAA

The copolymers of PMETAC-*co*-PAPMAA were synthesized by free-radical polymerization. Briefly, METAC (160 mg), APMAA (50 mg), and ACVA (10 mg) were dissolved in 2 mL of a 50% (*v*/*v*) EtOH–water mixture. After degassing with nitrogen for 10 min, polymerization was allowed to proceed for 24 h at 60 °C. The resulting PMETAC-*co*-PAPMAA copolymer was transferred into a pre-swollen membrane tube (Spectra/Por; molecular-weight cutoff size: 3500), dialyzed for 24 h against 2 L water, which was changed after 2, 5, and 8 h, and then was freeze-dried. The yield of the obtained polymer was 42.4% (89.5 mg).

### 3.3. Preparation of PMETAC-co-PAPMAA(HpD)

After 40 mg of the obtained PMETAC-*co*-PAPMAA was weighed into a 10 mL flask, 1 mL of phosphate buffer solution (pH 7.0) containing WSCD-HCl (15.5 mg), NHS (11.5 mg), and HpD (2.5 mg) was added to the flask and stirred for 20 h at 25 °C. The mixture was transferred into a pre-swollen membrane tube (Spectra/Por; molecular-weight cutoff size: 3500) and dialyzed for 24 h against 2 L water, which was changed after 2, 5, and 8 h, followed by freeze-drying. The yield of the obtained polymer was >99% (41.0 mg).

### 3.4. Characterizations

SEC measurements were performed using a Shimadzu HPLC system (Model no. LC-20AD, Kyoto, Japan) equipped with TSK gel columns (G3000PW and G5000PW) (Tosoh, Minato-Ku, Tokyo, Japan), an RID-10A refractive index detector, and an RF-10A_XL_ fluorescence detector (Shimadzu, Kyoto, Japan). Elution was performed with 0.5 M acetic acid aqueous solution containing tetramethylammonium hydroxide pentahydrate (28 mM) at a flow rate of 0.5 mL min^−1^ at 25 °C. The fluorescence at an excitation/emission wavelength of 400/635 nm was recorded using an RF-10A_XL_ fluorescence detector. ^1^H NMR spectra were recorded at room temperature on a JEOL-400YH spectrometer at 400 MHz (JEOL, Tokyo, Japan) using D_2_O. The UV–vis spectra were collected using a DS-11 spectrophotometer (DeNovix, Wilmington, DE, USA). The fluorescence spectra were collected on an F-7000 fluorescence spectrophotometer (HITACHI, Tokyo, Japan).

### 3.5. Cytotoxicity of PMETAC-co-PAPMAA(HpD)

RGM1 cells, a gastric epithelial cell line, were obtained from RIKEN BioResource Center (Tsukuba, Japan) [19]. RGM1 cells were cultured in Dulbecco’s modified Eagle’s medium (DMEM) containing 10% fetal bovine serum (FBS), 100 units/mL penicillin, and 100 µg/mL streptomycin at 37 °C in a humidified 5% CO_2_ atmosphere. RGM-1 cells were seeded on a 96-well plate at a density of 1 × 10^4^ cells/well and incubated overnight in 100 μL of culture medium. After the medium was changed to 90 μL of fresh DMEM, 10 µL of the PMETAC-*co*-PAPMAA(HpD) solution of different concentrations was added to each well at different concentrations. After incubation for 24 h, 10 μL of WST-8 reagent (Cell Counting Kit-8, Dojindo, Kamimashiki-gun, Kumamoto, Japan) was added to each well. The absorbance at 450 nm was measured on a DTX880 multi-mode microplate reader (Beckman Coulter, Brea, CA, USA), and the cellular viability was calculated relative to the non-treated cells. The mean and standard deviation were calculated from four independent experiments.

### 3.6. Animals

Four-week-old male Wistar rats (Charles River Laboratories, Inc., Atsugi, Kanagawa, Japan) were used in all experiments. The animals were housed three per cage and provided with water and rat chow *ad libitum*. The animals were maintained in a standard 12 h light–dark cycle.

### 3.7. Marking with Tissue-Adhesive Porphyrin and Hematoporphyrin

The rats were randomly divided into two groups: HpD and PMETAC-*co*-PAPMAA(HpD). They were fasted for 10 h and placed in the supine position; their abdomen was shaved and prepared with povidone–iodine solution. After sedation with an anesthesia with medetomidine-midazolam and butorphanol, they were disinfected, and the stomach was exposed through a midline incision in the cardiac fossa (Figure S1). An amount of 10 μL of each drug, 200 μM of HpD, and 5 mg/mL of PMETAC-*co*-PAPMAA(HpD) including same HpD concentration, were injected into the anterior wall of the stomach. After 1, 3, and 7 days under sedation with the anesthesia with medetomidine-midazolam and butorphanol, the stomach was exposed through a midline incision in the cardiac fossa and the fluorescent images were captured under UV irradiation at 375 nm using an LED lamp and a Nikon D5 camera (Nikon, Tokyo, Japan) equipped with a red gelatin filter, as shown in Figure S2.

To quantify the red fluorescence intensity of porphyrin in the stomach tissue, the stomach was resected, opened along the greater curvature, and stretched on a Styrofoam board covered with black sheets for quantitative analysis (Figure S2). Fluorescent images were captured under UV irradiation at 375 nm using an LED lamp and a Nikon D5 camera (Nikon, Minato-ku, Tokyo, Japan) equipped with a red gelatin filter. The emission spectrum of the LED lamp is shown in Figure S3, which exhibits a peak at 375 nm. The captured fluorescence images were analyzed using ImageJ software (National Institutes of Health, Bethesda, MD, USA). The area emitting red fluorescence was selected as the region of interest (ROI), and its mean fluorescence intensity was measured. The mean and standard error of the mean (SEM) were calculated from three independent experiments.

### 3.8. Safety Evaluation

Content of mammalian body fluid is almost 80% of body weight, and total volume of body fluid in rats weighting 200 g is approximately 160 mL. Since we injected aHpD (5 mg/mL, 100 μL) intraperitoneally into the rats weighting 200 g, final concentration of the aHpD was maximally 3.2 μg/mL even when the aHpD was absorbed into the systemic fluid. From this calculation, we expected the low toxicity of aHpD in vivo.

To confirm the safety of aHpD, the rats were randomly divided into PBS-, HpD-, and PMETAC-*co*-PAPMAA(HpD)-treated groups. Body weight was measured, and blood samples were collected from the tail vein and placed in a microtube containing sodium heparin before and on days 1 and 7 after intraperitoneal injection of 100 µL of PBS, HpD (200 μM), and PMETAC-*co*-PAPMAA(HpD) (5 mg/mL). Plasma samples were obtained by centrifugation (6200 rpm, 2000 g for 10 min) of blood. Plasma samples were assayed for ALT, AST, BUN, and CRE using an automatic animal biochemistry analyzer Fuji Drychem 7000 V (Fuji Film Co., Tokyo, Japan). On day 7 after the intraperitoneal injection, an abdominal incision was made to check for organ adhesions between the intestine and the peritoneum.

### 3.9. Visibility Test

To evaluate the visibility of tissue marking agents, India ink, and our developed PMETAC-*co*-PAPMAA(HpD) in the tissue, fresh porcine colon (JA ZEN-NOH Meat Foods Co., Ltd., Shimotsuma, Ibaraki, Japan) was employed as a model tissue. One hundred microliters of India ink or PMETAC-*co*-PAPMAA(HpD) (5 mg mL^−1^) was injected into the submucosa from the mucosal side, and the visibility of the marking agent was observed from the serosal side. In the case of PMETAC-*co*-PAPMAA(HpD), the visibility of the marking agent was observed from the serosal side under UV irradiation at 375 nm.

### 3.10. Statistical Analysis

Differences between the two groups were examined for statistical significance using a Student’s *t*-test. Differences between more than three groups were examined for statistical significance using one-way ANOVA followed by Tukey’s test (SPSS software; IBM Corp, Armonk, NY, USA). A *p* value of <0.05 was considered significant for all statistical analyses.

## 4. Conclusions

In summary, in this study, we designed and developed tissue-adhesive polymeric materials with long-term fluorescent tissue-marking ability. Tissue-adhesive porphyrin remained in the tissue of the rat stomach wall for more than 1 week owing to the introduction of tissue-adhesive polycations consisting of quaternary ammonium salt groups. In addition, even an injection of PMETAC-*co*-PAPMAA(HpD) into the abdominal cavity showed no toxicity or abnormal organ adhesion. This may be due to the elimination of PMETAC-*co*-PAPMAA(HpD) from the body, because the molecular weight of PMETAC-*co*-PAPMAA(HpD) was approximately 20 kDa. These results indicate that PMETAC-*co*-PAPMAA(HpD) is a promising tissue-adhesive material for tissue marking.

## Data Availability

Not applicable.

## Supplementary Materials

The following supporting information can be downloaded at: https://www.mdpi.com/article/10.3390/ijms23084218/s1.
