# Supplementary material for: Long-Term Fluorescent Tissue Marking Using Tissue-Adhesive Porphyrin with Polycations Consisting of Quaternary Ammonium Salt Groups
Source: Int J Mol Sci. 2022 Apr 11;23(8):4218. doi: 10.3390/ijms23084218 (PMC9029083; doi:10.3390/ijms23084218)
Supplement: Supplementary file 1 [file ijms-23-04218-s001.zip › ijms-1668446-supplementary.pdf]

Supplementary Information

Long-term fluorescent tissue marking using tissue-adhesive porphyrin with polycations consisting of quaternary ammonium salt groups

Yoshiki Komatsu[1,†], Toru Yoshitomi[2,†,*], Kinji Furuya[3], Takafumi Ikeda[1], Azusa Terasaki[4], Aoi Hoshi[1], Naoki Kawazoe[2], Guoping Chen[2], and Hirofumi Matsui[5,*]

[1] Graduate School of Comprehensive Human Sciences, University of Tsukuba, Ibaraki 305-8577, Japan

[2] Research Center for Functional Materials, National Institute for Materials Science, 1-1 Namiki, Tsukuba, Ibaraki, 305-0044 Japan

[3] Department of Gastrointestinal and Hepato-Biliary-Pancreatic Surgery, Faculty of Medicine, University of Tsukuba, Tsukuba, Ibaraki 305-8575, Japan

[4] Division of Breast and Endocrine Surgery, Faculty of Medicine, University of Tsukuba, Ibaraki 305-8577, Japan

[5] Division of Gastroenterology, Faculty of Medicine, University of Tsukuba, Ibaraki 305-8575, Japan

[†]Equal contribution

* Author to whom correspondence should be addressed.

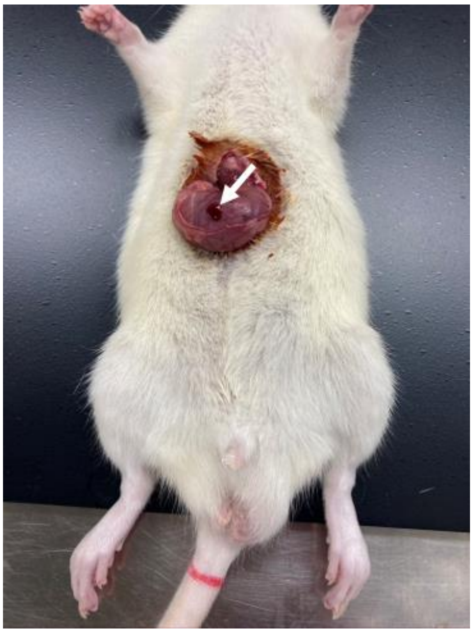

Figure S1. Photograph of the exposed stomach through a midline incision in the cardiac fossa. White arrow indicates the injection site of tissue-adhesive porphyrin.

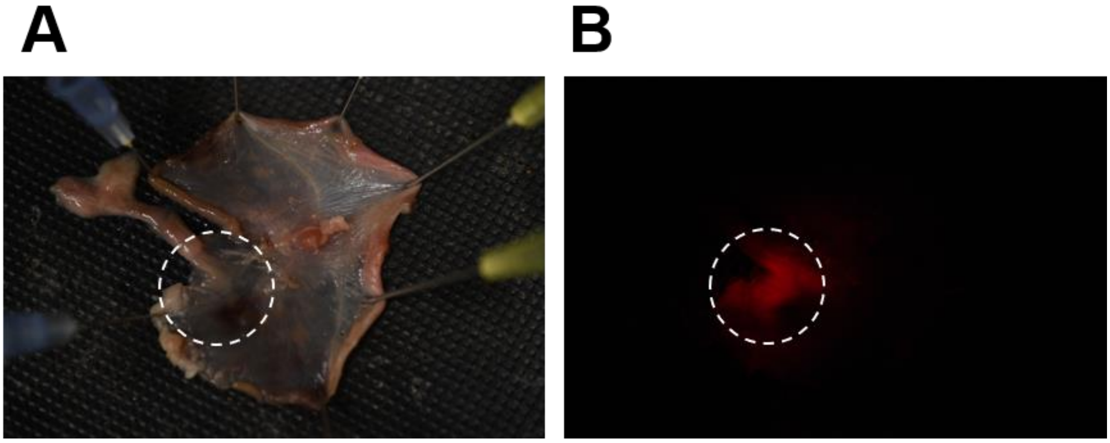

Figure S2. Fluorescent detection of PMETAC-*co*-PAMPAA(HpD) on the exposed stomach at 7 days after local injection of PMETAC-*co*-PAMPAA(HpD) into the anterior wall of the stomach of rats. (A) Photo of the stomach opened on the side of the curvature under the white light by digital camera. (B) Photo of the stomach opened on the side of the curvature under the irradiation at 375 nm by LED light by digital camera with equipped with a red gelatin filter. White dot circles show the localization of marking. Image analysis was performed using Image J.

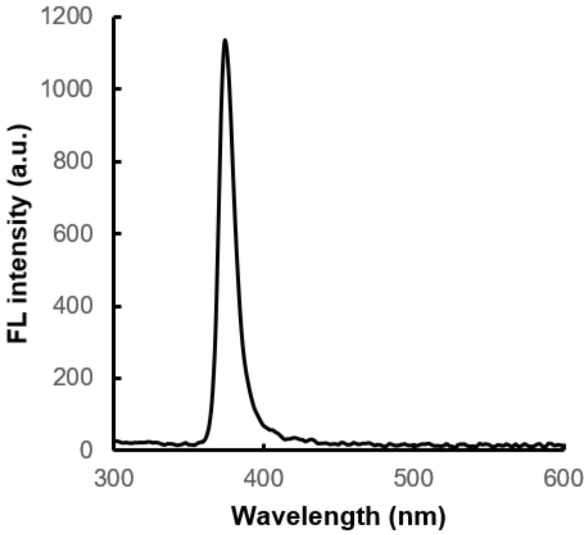

Figure S3. Emission spectrum of a LED lamp
